# Reconstruction of phyletic trees by global alignment of multiple metabolic networks

**DOI:** 10.1186/1471-2105-14-S2-S12

**Published:** 2013-01-21

**Authors:** Cheng-Yu Ma, Shu-Hsi Lin, Chi-Ching Lee, Chuan Yi Tang, Bonnie Berger, Chung-Shou Liao

**Affiliations:** 1 Department of Computer Science, National Tsing Hua University, Hsinchu, Taiwan; 2 Institute of Bioinformatics and Structural Biology, National Tsing Hua University, Hsinchu, Taiwan; 3 Bioinformatics Center, Chang Gung University, Taoyuan, Taiwan; 4 Department of Computer Science and Information Engineering, Providence University, Taichung, Taiwan; 5 Computer Science and Artificial Intelligence Laboratory, Massachusetts Institute of Technology, Cambridge, MA 02139, USA; 6 Department of Mathematics, Massachusetts Institute of Technology, Cambridge, MA 02139, USA; 7 Department of Industrial Engineering and Engineering Management, National Tsing Hua University, Hsinchu, Taiwan

## Abstract

**Background:**

In the last decade, a considerable amount of research has been devoted to investigating the phylogenetic properties of organisms from a systems-level perspective. Most studies have focused on the classification of organisms based on structural comparison and local alignment of metabolic pathways. In contrast, global alignment of multiple metabolic networks complements sequence-based phylogenetic analyses and provides more comprehensive information.

**Results:**

We explored the phylogenetic relationships between microorganisms through global alignment of multiple metabolic networks. The proposed approach integrates sequence homology data with topological information of metabolic networks. In general, compared to recent studies, the resulting trees reflect the living style of organisms as well as classical taxa. Moreover, for phylogenetically closely related organisms, the classification results are consistent with specific metabolic characteristics, such as the light-harvesting systems, fermentation types, and sources of electrons in photosynthesis.

**Conclusions:**

We demonstrate the usefulness of global alignment of multiple metabolic networks to infer phylogenetic relationships between species. In addition, our exhaustive analysis of microbial metabolic pathways reveals differences in metabolic features between phylogenetically closely related organisms. With the ongoing increase in the number of genomic sequences and metabolic annotations, the proposed approach will help identify phenotypic variations that may not be apparent based solely on sequence-based classification.

## Background

One of the major challenges in biology is to reconstruct phyletic relationships between living organisms. Various phylogenetic inference methods have been proposed to unravel this critical problem by using genomic data [1]; different phylogenetic trees have been reconstructed based on the similarity of sequences of genes encoding 16S ribosomal RNAs [2] and other marker genes [3-5].

With the increasing availability of whole-genome sequences, proteomic data, and annotated metabolic reactions, more homologous characters between different organisms can be identified to infer phylogenetic trees. In addition to genomic comparisons, a number of recent studies have begun to explore phylogenetic distance between species based on metabolic properties, either alone or in combination with sequence features [6-17]. Conserved metabolic pathways have been used to explicitly derive phylogenetic trees through a variety of approaches. For example, Forst *et al. *measured distances between organisms by iteratively aligning enzymes based on sequence similarities [6]. Heymans *et al. *conducted a pairwise comparison of a single common metabolic pathway between organisms to build phylogenetic trees; they created a distance matrix based on topological relationships among enzymes (reaction graph) [7]. Clemente *et al. *hierarchically compared EC (Enzyme Commission) numbers of a common metabolic pathway among multiple organisms to measure pathway similarity [9]. All these studies, however, only compared a single metabolic pathway independently when retrieving metabolic network information.

Subsequently, Clemente *et al. *extended the EC-based classification method to compare all the common metabolic pathways between multiple species [13]. On the other hand, Oh *et al. *used a machine learning approach for computing a distance metric using an exponential graph kernel based on nine common pathways [11]. Another way to compare a pair of metabolic pathways between organisms is to use topological properties to define the existence/absence of metabolic pathways among organisms [12]; it is thus a network comparison-based method. Mazurie *et al. *used descriptors of structure and complexity of metabolic reactions to calculate phylogenetic distances [14]. Borenstein *et al. *devised a seed approach based on essential metabolites to carry out large-scale reconstruction of phylogenetic trees [15]. Recently, Chang *et al. *proposed an approach from the perspective of enzyme substrates and corresponding products in which each organism is represented as a vector of substrate-product pairs, and the vectors are then compared to reconstruct a phylogenetic tree [17]. Furthermore, Mano *et al. *considered the topology of pathways as chains and used the pathway alignment method developed by Pinter *et al. *[10] to classify species [16]. Although comparison and alignment of metabolic networks have been applied to reconstruct phyletic relationships [9,10,12-16], previous studies only considered pairwise structural comparison of conserved metabolic pathways in a local fashion.

Network alignment has become central to systems biology; it can be divided into two types: local and global alignment. Local network alignment is defined as an alignment of small subnetworks from one network with one or more subnetworks in another network. Because such alignments allow one node to have different pairings in different subnetworks, local network alignment may generate ambiguous results. On the other hand, global network alignment can provide a one-to-one mapping for all nodes between networks. That is, the aim is to find multiple independent regions of localized network similarity. Global alignment of multiple networks provides clusters across species that best represent conserved biological functions. Therefore, to investigate phyletic relationships from metabolic networks, we selected IsoRankN [18], a global multiple-network alignment tool that simultaneously integrates sequence information with topological properties to cluster functionally similar proteins across species.

## Results

We used IsoRankN to generate a biologically relevant multipartite mapping between organisms. The clusters of enzymes across the networks in the mapping derived by IsoRankN represent conserved biological reactions and functions. We adapted an entropy measure [18] as the filtering criterion to remove non-consistent enzyme clusters (see Methods). To construct a phyletic tree comprising multiple species, we defined a pairwise distance measure between two organisms. Data for all the metabolic networks and the enzyme sequences used in this study were retrieved from the KEGG database [19]. Additional file 1 lists information for the organisms we tested.

First, we classified 26 organisms at the phylum scale and compared our results with recent studies. Moreover, the approach was applied to phylogenetically closely related organisms to reconstruct phyletic relationships concerning specific metabolic characteristics, such as the light-harvesting systems between *Prochlorococcus *and *Synechococcus *groups, fermentation types between *Lactobacillus*, and sources of electrons used for photosynthesis between green sulfur and green nonsulfur bacteria.

### Phylum-scale classification

**Figure 1:**
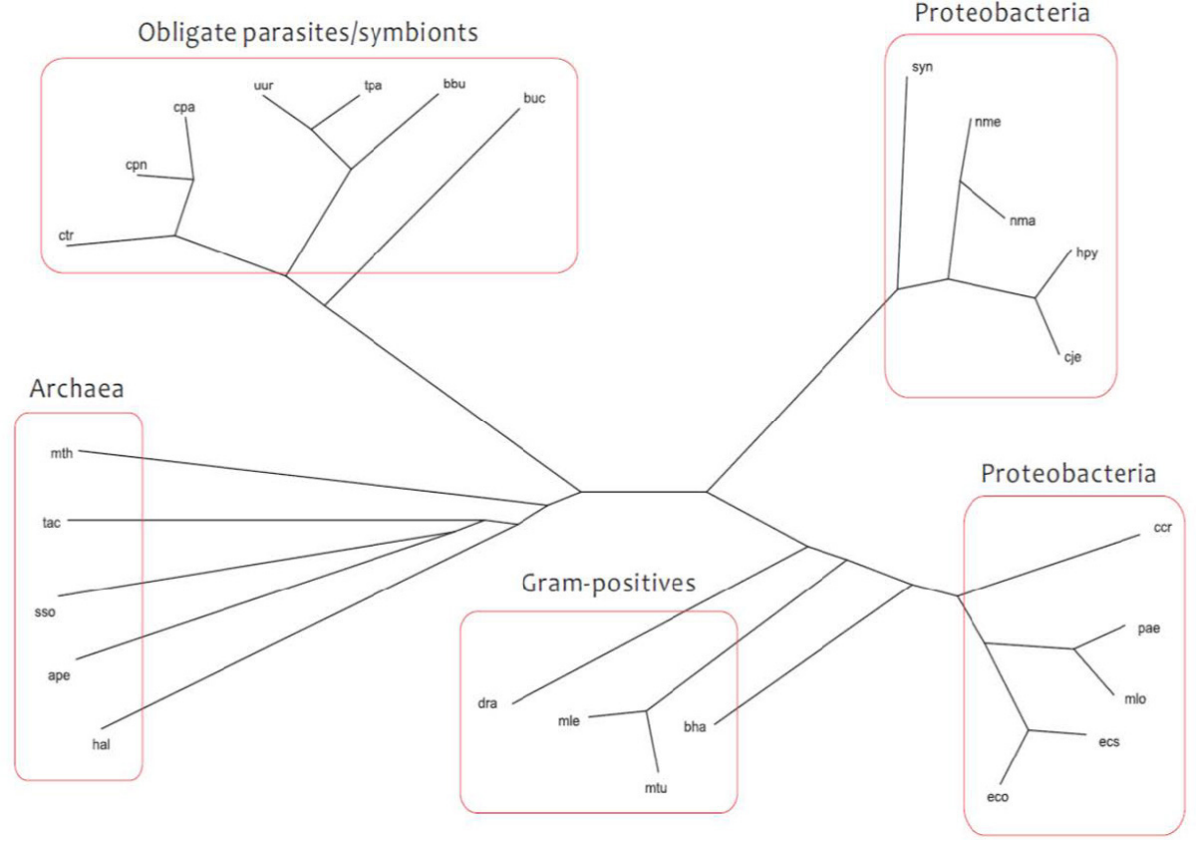
**Phylum-scale classification**. Our reconstruction of a phyletic tree consisting of 26 organisms; the tree was drawn with Dendroscope [33].

**Figure 2:**
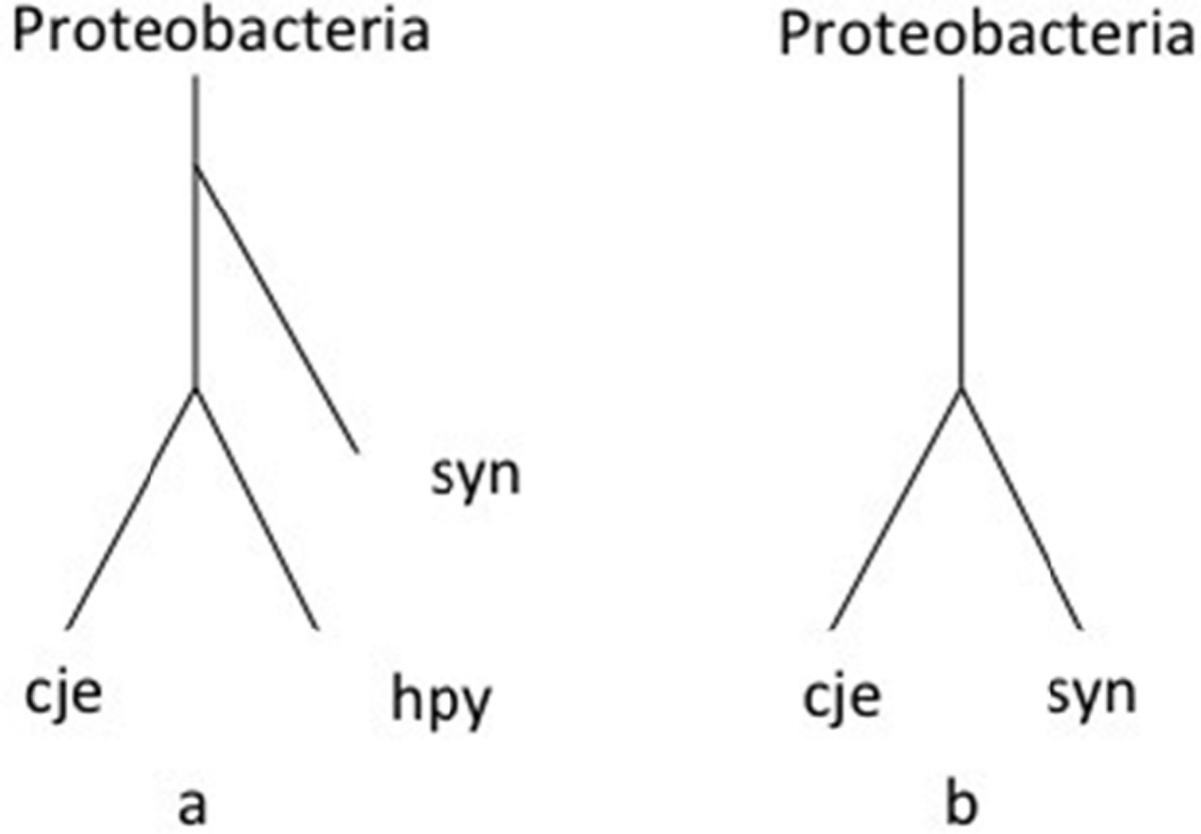
**Differences between our tree and the tree generated by Zhang et al**. (a) In our tree, cje and hpy are grouped together because they both belong to ε-proteobacteria. (b) In the study of Zhang *et al*., cje and syn are clustered together, and buc and hpy are grouped into the category obligate parasites/symbionts. cje, *Campylobacter jejuni subsp. jejuni *NCTC 11168; hpy, *Helicobacter pylori *26695; syn, *Synechocystis sp*. PCC 6803;.

Following recent work through the pathway comparison-based approach [12] and substrate-product relationships [17], we chose 26 prokaryotes belonging to four categories: archaea, Gram-positive bacteria, obligate parasites/symbionts, and Proteobacteria (Additional file 1: Phylum scale). Our method correctly divides the 26 organisms into the four groups (Figure 1). In general, the classification result is similar to that derived from each of the two recent approaches (Additional file 2). Upon detailed comparison of tree topologies, the different relative positions can be explained as follows. To clarify the differences between our reconstruction and that generated by the network comparison-based approach of Zhang *et al. *[16], we consider the three organisms *Buchnera aphidicola *APS (buc), *Campylobacter jejuni subsp. jejuni *NCTC 11168 (cje), and *Helicobacter pylori *26695 (hpy). With our method, hpy and cje were appropriately grouped together in the same subtree of the category Proteobacteria as in the NCBI taxonomy [20] (Figure 2). On the other hand, hpy and buc were grouped together in the category obligate parasites/symbionts in Zhang *et al*.'s reconstruction (Figure 2) [16]. Because the pathway comparison-based method only considers the diameter of pathways and the average length of the shortest paths within pathways as topological features, the approach lacks sufficient network information and therefore cannot reveal all of the relevant metabolic properties.

The above result shows that our method can correctly classify organisms into main categories. For the cases shown below, we tested our method with consideration of specific metabolic features.

### Lactobacillus

**Figure 3:**
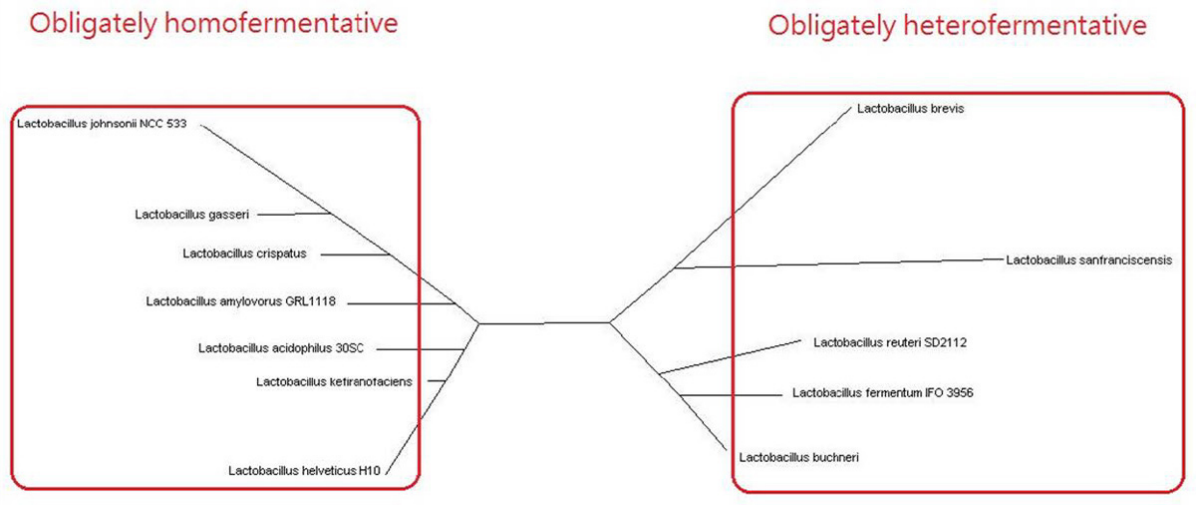
**Lactobacillus**. Based on different sugar fermentation patterns, 12 *Lactobacillus *species can be divided into two groups: obligately homofermentative and obligately heterofermentative metabolism.

We assessed 12 species of *Lactobacillus*, which is a genus of Gram-positive lactic acid bacteria that have limited biosynthetic capacity and thus are restricted to environments in which sugars are present. With reference to known sugar fermentation patterns [21,22], our approach could successfully divide 12 *Lactobacillus *species into two broad metabolic categories: obligately homofermentative and obligately heterofermentative metabolism (Figure 3). This classification is similar to previous studies based on proteomics [23], a rRNA dataset [24,25], and marker genes [26]. The difference between these two categories at the enzyme level possibly comes from the presence or absence of key cleavage enzymes in the glycolysis pathway and phosphoketolase pathway [22].

### *Prochlorococcus *and *Synechococcus*

**Figure 4:**
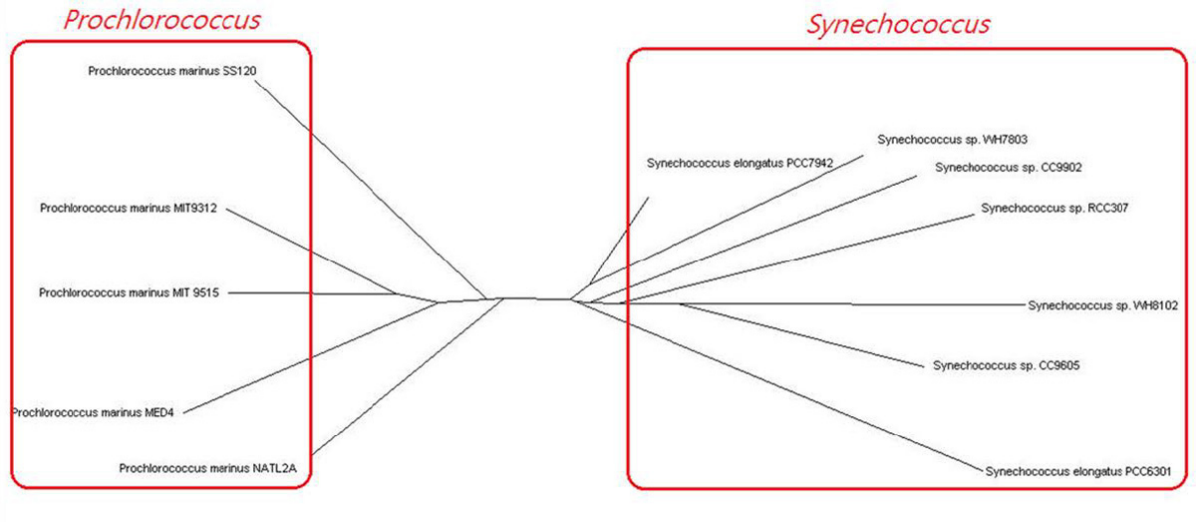
***Prochlorococcus *and *Synechococcus***. Global alignment of multiple metabolic networks separates *Prochlorococcus *and *Synechococcus *into two groups and reveals differences between light-harvesting systems.

Next, we selected 12 organisms from *Prochlorococcus *and *Synechococcus*. These two genera show greater than 96% similarity in their 16S rRNA sequences; however, they have different light-harvesting systems. *Prochlorococcus *has divinyl chlorophyll a (chl a2), monovinyl and divinyl chlorophyll b (chl b) as its major photosynthetic pigments, but *Synechococcus *has chlorophyll a (chl a) and phycobiliproteins that are typical of cyanobacteria [27]. In addition to these differences in light-harvesting systems, their utilization of nitrogen sources also differs [27,28]. Compared with conventional reconstruction methods based on 16S rRNA information, our method could more correctly divide them into two groups and revealed differences in their metabolic features (Figure 4).

### Green sulfur and green nonsulfur bacteria

**Figure 5:**
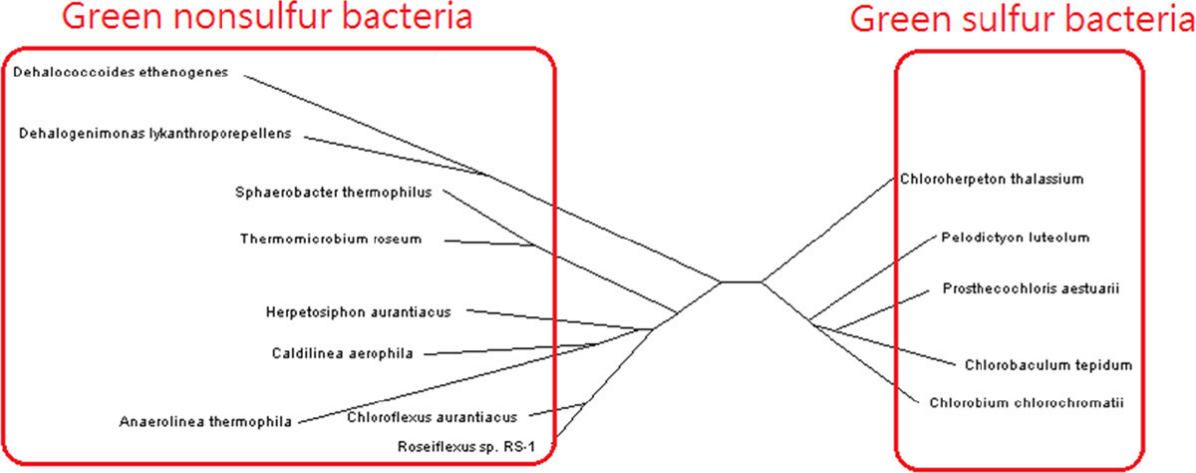
**Green sulfur and green nonsulfur bacteria**. Anaerobic photoautotrophic bacteria can be classified into two groups: green sulfur group and green nonsulfur group.

In our final experiment, we tested our method on green sulfur and green nonsulfur bacteria from anaerobic photoautotrophic bacteria. These organisms use two different sources of electrons in photosynthesis. Green sulfur bacteria use sulfide ion as the electron donor, whereas green nonsulfur bacteria do not [29]. We reconstructed a phyletic tree for 14 species (Figure 5); our classification result clearly reflects this metabolic characteristic. The green sulfur and green nonsulfur species were classified into two different groups; phylum *Chloroherpeton*, *Pelodictyon*, *Prosthecochloris*, *Chlorobaculum *and *Chlorobium *are in green sulfur group, whereas the other nine strains in different phyla are classified into green nonsulfur group. The result implies that the proposed method can identify unique metabolic features.

Based on global alignment of multiple metabolic networks, our approach can classify organisms into main categories that reflect living style and phenotypes. The above cases clearly show that the resulting phyletic trees reflect specific metabolic characteristics among species. Thus, our approach can provide phyletic reconstructions at high resolution and characterize differences in metabolic features between phylogenetically closely related organisms.

## Methods

We employed IsoRankN to explore functional similarities and differences in multiple metabolic networks. The key idea of IsoRankN is briefly introduced (Additional file 3), and a detailed description has been published in [18]. IsoRankN is a global multiple-network alignment tool based on spectral clustering methods. Given several metabolic networks, in which the enzymes and metabolites are represented as nodes and the reactions catalyzed by enzymes are represented as edges in each network, the algorithm first computes pairwise functionally similar scores between all the cross-species enzymes [30]. The next step uses the concept of the star alignment approach and personalized spectral clustering. In addition, we also used the functional consistency measure [18] to further refine the clusters obtained by IsoRankN.

To remove non-consistent enzyme clusters, we adapted an entropy measure *S_V _*is used as the consistency measure, which represents the degree of functional uniformity of enzymes in each cluster.

$$\text{H}\left(S_{V}\right)=\text{H}\left(p_{1},p_{2},\cdots p_{d0}\right)=-\sum_{i=1}^{d}p_{i}\log p_{i}$$

where *p_i _*is the fraction of *S_V _*with KEGG group ID i. A cluster with lower entropy implies greater within-cluster consistency with respect to KEGG annotations, and thus we select the clusters with lower entropy to extract a greater amount of information on the phylogenetic relationships between the test organisms.

A phyletic tree comprising multiple species is reconstructed based on a distance measure defined by the fraction of the identified clusters in which the constituent enzymes appear in the two organisms. The distance between two organisms A and B is defined as follows: $\frac{\left|S_{A\cap B}\right|}{\left|S_{A\cup B}\right|}$ where |*S*_*A*∩*B*_| denotes the number of clusters that contain enzymes in both organisms *A *and *B*, and |*S*_*A*∪*B*_| denotes the number of clusters in which the constituent enzymes are in either organism *A *or *B*. We remark that only the clusters with lower *mean entropy *are considered. The *mean entropy *of a cluster measures its functional consistency, and as noted above, lower entropy implies greater within-cluster consistency with respect to KEGG annotations. Thus, to obtain consistency with respect to sequence-based KEGG annotation and topological features, we select the clusters having entropy no larger than 0.5.

Based on the above process, a distance matrix can be obtained. We then used PHYLIP [32] to build a phyletic tree based on the distance matrix. The visualization tool, Dendroscope [33], was used to display the phyletic trees. All experiments were performed on a platform consisting of Intel(R) Xeon(R) CPU E31230 (3.20 GHz, 16 GB memory) machines running the Linux system.

## Discussion

Establishing network alignments is critical in evolutionary and systems biology [34]. Several approaches to multiple network alignment have been developed to infer the global homologous characters between complete networks; these approaches include Græmlin [35,36], NetworkBLAST-M [37], IsoRank [30], IsoRankN [18], GRAAL [38], and SubMAP [39]. Græmlin is a machine learning approach implemented by initially using sequence features and then incorporating local network information. However, it is difficult to select training data for reconstructing phyletic relationships between close organisms [35]. NetworkBLAST-M is a local network alignment tool, which cannot reveal complete topological information. Kuchaiev *et al. *developed the pairwise sequence-free global network alignment tool, GRAAL, with which they defined a distance metric between two species by using the edge correctness ratio of pairwise metabolic network alignment results and reconstructed phylogenetic trees [38]. Because the tool only considers topological information of metabolic networks, the sequence features that are ignored may play important biological roles in phylogeny. The first global network alignment algorithm, IsoRank, uses a spectral graph algorithm to measure an alignment between two networks based on both sequence similarity between nodes and topological similarity of their neighborhoods. Ay *et al. *extended the idea of the IsoRank algorithm for pairwise network alignment to metabolic networks but did not consider multiple network alignment [39]. Therefore, for our purpose we selected IsoRankN, a global multiple network alignment tool that simultaneously integrates sequence information with topological properties to cluster functionally similar proteins across species. Liao *et al. *[18] demonstrated that IsoRankN outperformed existing algorithms for global multiple network alignment of protein interaction networks with respect to coverage and consistency.

**Figure 6:**
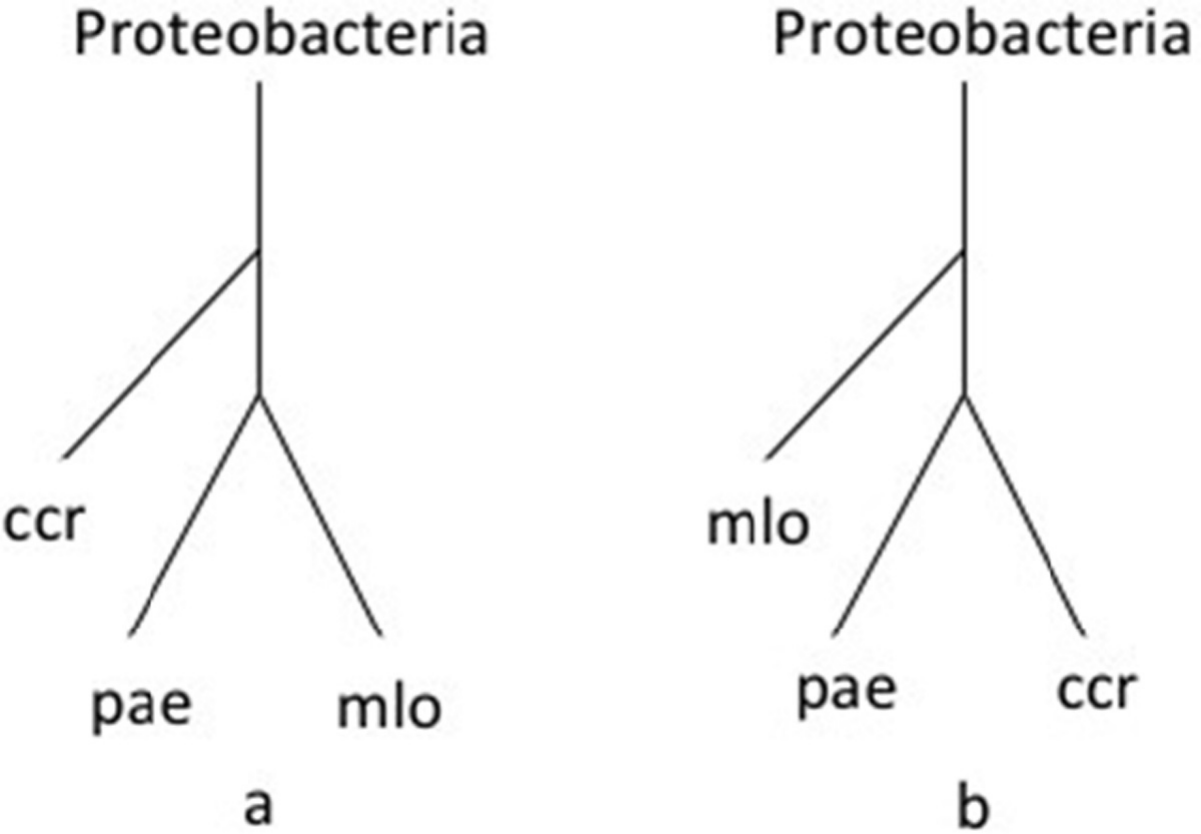
**Differences between our tree and the tree generated by Chang et al**. (a) In our tree, pae is closer to mlo than ccr because pae and mlo have two highly similar pathways. (b) In the study of Chang *et al*., pae is closer to ccr than to mlo. ccr, *Caulobacter crescentus *CB15; mlo, *Mesorhizobium loti *MAFF303099; pae, *Pseudomonas aeruginosa *PAO1;.

Recall our first reconstruction result on the 26 prokaryotic organisms (Figure 1). Note that our phyletic classification is quite similar to the reconstruction of Chang et al. [17], although there are certain differences (Additional file 2). We try to investigate the difference through a new quantitative analysis method. Because networks that are similar share a greater number of common enzymes, for each KEGG pathway ID we computed the number of constituent enzymes associated with this ID in the clusters obtained from IsoRankN for a pair of organisms. This method is used to evaluate functionally similar pathways between those two organisms. We applied the method to assess phylum-scale reconstruction and compared with the results of Chang et al. to find more subtle phenotypic differences. With a detailed comparison of tree topologies, we then consider the instance of three organisms: *Caulobacter crescentus *CB15 (ccr), *Mesorhizobium loti *(mlo) and *Pseudomonas aeruginosa *PAO1 (pae). pae is closer to mlo than to ccr in our tree (Figure 6a). In the reconstruction of Chang et al. [17], however, pae is closer to ccr than to mlo (Figure 6b). According to the statistics of the KEGG pathways for the three species pairs, namely (mlo, pae), (ccr, mlo), and (ccr, pae), two pathways, ko00260 and ko00860 for the pair (mlo, pae), show more functional similarity than those for the pairs (ccr, mlo) and (ccr, pae) (Additional file 4). The quantitative analysis demonstrates that pae and mlo have stronger phenotypic similarity.

As for phylogenetically closely related organisms, we then applied the same analysis to *Lactobacillus*. For our reconstruction (see Figure 3), we consider three pairs of organisms with high 16S rRNA sequence similarity: *Lactobacillus gasseri *(lga) versus *Lactobacillus johnsonii *NCC 533 (ljo), *Lactobacillus fermentum *IFO 3956 (lfe) versus *Lactobacillus reuteri *SD2112 (lru), and finally lfe versus lga. The former two pairs come from the same groups, respectively, and the last pair was selected from different groups in our reconstruction. As shown in Additional file 5, the pair (lga, ljo) in the homofermentation group shares more enzymes than those for the pair (lfe, lga) from different groups according to the statistics of the KEGG pathways (Additional file 5a); similarly, (lfe, lru) has more common enzymes than those for (lfe, lga) (Additional file 5b). That is, *Lactobacillus *species in the same group in our classification show more functional similarity than those species from different groups. More precisely, concerning the glycolysis/gluconeogenesis pathway, ko00010, (lga, ljo) and (lfe, lru) share more constituent enzymes than those for (lfe, lga). These results show that our reconstruction can reveal specific metabolic features.

We also analyzed species from *Prochlorococcus *and *Synechococcus*, which have different light-harvesting systems. For our reconstruction (see Figure 4), we consider three pairs of organisms: *Prochlorococcus marinus *SS120 (pma) versus *Prochlorococcus marinus *MIT 9515 (pmc), *Synechococcus sp*. WH8102 (syw) versus *Synechococcus sp*. WH7803 (syx), and finally pma versus syx. The former two pairs come from the same groups, respectively, and the last one was selected from different groups in our reconstruction. However, there is no obvious difference when we compare (pma, pmc) and (syw, syx) with (pma, syx) (Additional file 6a and 6b). In such a case, the quantitative analysis cannot explicitly classify the species with high sequence similarity regarding their particular metabolic features.

In contrast, our classification by using global alignment of multiple metabolic networks can successfully determine phenotypic similarity (Figure 4). Because our approach incorporates topology features of metabolic networks with sequence similarity, it affords a more in-depth analysis of the phyletic reconstruction.

## Conclusions

Most studies have focused on the classification of organisms based on structural comparison and local alignment of metabolic pathways. In contrast, global alignment of multiple metabolic networks, which compensates sequence-based phylogenetic analyses, may provide more comprehensive information. Therefore, we propose a new approach that uses the global network alignment tool, IsoRankN, to reconstruct phyletic relationships of multiple species. Our phyletic trees lie between conventional genotypic construction and phenotypic reconstruction. We demonstrated that our reconstruction has the capacity to explore more in-depth metabolic features and subtle phenotypic differences, such as light-harvesting systems, fermentation type, and sources of electrons for photosynthesis.

The growing mass of systems-level data allows our approach to find more applications to identify phenotypic variations hidden behind sequence-based classification [1,40]. In addition to metabolic network information, Suthram *et al. *[41] showed that phylogenetic relationships may be inferred from protein interaction networks. They identiﬁed conserved species-speciﬁc complexes in protein interaction networks and built a phylogenetic tree based on the complexes because interactions between proteins may imply conservation of speciﬁc groups. Although false-positives exist in protein-protein interaction data, comparative analysis of protein-protein interaction networks of closely related organisms can reveal phenotypic properties [42]. Therefore, global alignment of multiple protein-protein interaction networks may provide a high-resolution look at phyletic reconstruction. It is worthwhile to explore the phenotypic differences between global network alignment of multiple metabolic networks and protein interaction networks. In the future, better quantitative and qualitative analyses of metabolic pathways between organisms would also be of interest.

## Competing interests

The authors declare that they have no competing interests.

## Authors' contributions

CYM and CSL conceived, designed, and performed this study. CYM conducted research under supervision of CSL and CYT. CYM, SHL and CCL designed the experiments. CYM, SHL, CCL, CYT, BB and CSL wrote the manuscript. All the authors read and approved the final manuscript.

## Declarations

This work was funded in part by the National Science Council of Taiwan under the Grants NSC100-2221-E-007-108-MY3 (to C.-S.L.) and NSC100-2221-E-126-011-MY3 (to C.Y.T), NIH Grant GM081871 (to B.B.) and MOE Grant 101N2074E1 (to C.-S.L.). The publication costs for this article were funded by the National Science Council of Taiwan.

This article has been published as part of *BMC Bioinformatics *Volume 14 Supplement 2, 2013: Selected articles from the Eleventh Asia Pacific Bioinformatics Conference (APBC 2013): Bioinformatics. The full contents of the supplement are available online at http://www.biomedcentral.com/bmcbioinformatics/supplements/14/S2.

## Supplementary Material

Additional file 1 **Organisms used in this study**. Edges represent the reactions catalyzed by enzymes in each metabolic network. All metabolic pathways were retrieved from KEGG [19]. Click here for file

Additional file 2 **Comparison of reconstructed phylogenic trees**. Left: Reconstruction by Chang *et al. *[17]. Right: Reconstruction by Zhang *et al. *[12]. Reprinted under the BioMed Central Open License agreement (BMC Bioinformatics). Click here for file

Additional file 3 **The IsoRankN algorithm**. Click here for file

Additional file 4 **Statistics for KEGG pathways between three pairs of organisms: (mlo, pae), (ccr, mlo) and (ccr, pae)**. The *x *axis represents KEGG pathway IDs, and the *y *axis represents the number of the constituent enzymes in the pathways. The two pathways ko00260 and ko00860 in the pair (mlo, pae) contain more functional orthologs than those in the pairs (ccr, mlo) and (ccr, pae). Click here for file

Additional file 5 **Statistics for KEGG pathways between two pairs of organisms in *Lactobacillus*: The *x *axis represents KEGG pathway IDs, and the *y *axis represents the number of the constituent enzymes in the pathways**. (a) (lga, ljo) in obligate homofermentation, and (lfe, lga) from different fermentation types. (b) (lfe, lru) in obligate heterofermentation, and (lfe, lga) from different fermentation types. Click here for file

Additional file 6 **Statistics for KEGG pathways between two pairs of organisms of *Prochlorococcus and Synechococcus*: The x axis represents KEGG pathway IDs, and the y axis represents the number of the constituent enzymes in the pathways**. (a) (pma, pmc) from *Prochlorococcus*, and (pma, syx) from *Prochlorococcus *and *Synechococcus*, respectively. (b) (syw, syx) from *Synechococcus*, and (pma, syx) from *Prochlorococcus *and *Synechococcus*, respectively. Click here for file
