# Supplementary material for: Reconstruction of phyletic trees by global alignment of multiple metabolic networks
Source: BMC Bioinformatics. 2013 Jan 21;14(Suppl 2):S12. doi: 10.1186/1471-2105-14-S2-S12 (PMC3549807; doi:10.1186/1471-2105-14-S2-S12)
Supplement: Additional file 1 — Organisms used in this study. Edges represent the reactions catalyzed by enzymes in each metabolic network. All metabolic pathways were retrieved from KEGG [19]. [file 1471-2105-14-S2-S12-S1.pdf]

Code	*Organism*	# of nodes	# of edges	Taxonomy
**Phylum scale**				
ape	*Aeropyrum pernix* K1	900	1429	Crenarchaeota
hal	*Halobacterium sp.* NRC-1	1005	1574	Euryarchaeota
mth	*Methanothermobacter thermautotrophicus* deltaH	906	1500	Euryarchaeota
sso	*Sulfolobus solfataricus P2*	1250	2172	Crenarchaeota
syn	*Synechocystis sp.* PCC 6803	1424	2088	Cyanobacteria
tac	*Thermoplasma acidophilum* DSM 1728	892	1334	Euryarchaeota
bha	*Bacillus halodurans* C-125	1625	3029	Firmicutes
dra	*Deinococcus radiodurans* R1	1376	2226	Deinococcus-Thermus
mle	*Mycobacterium leprae* TN	1090	1633	Actinobacteria
mtu	*Mycobacterium tuberculosis* H37Rv	1816	4546	Actinobacteria
bbu	*Borrelia burgdorferi* B31	379	448	Spirochaetes
buc	*Buchnera aphidicola* APS	618	779	γ-proteobacteria
cpa	*Chlamydophila pneumoniae* AR39	595	790	Chlamydiae
cpn	*Chlamydophila pneumoniae* CWL029	596	792	Chlamydiae
ctr	*Chlamydia trachomatis* D/UW-3/CX	584	776	Chlamydiae
tpa	*Treponema pallidum subsp. pallidum* Nichols	426	588	Spirochaetes
uur	*Ureaplasma parvum serovar 3* ATCC 700970	291	341	Tenericutes
ccr	*Caulobacter crescentus* CB15	1665	3015	α-proteobacteria
cje	*Campylobacter jejuni subsp. jejuni* NCTC 11168	995	1367	ε-proteobacteria
eco	*Escherichia coli K-12* MG1655	1887	3180	γ-proteobacteria
ecs	*Escherichia coli*	1862	3172	γ-proteobacteria

	O157:H7 Str. Sakai			
hpy	*Helicobacter pylori* 26695	889	1163	ε-proteobacteria
mlo	*Mesorhizobium loti* MAFF303099	2178	4738	α-proteobacteria
nma	*Neisseria meningitidis* Z2491	1079	1513	β-proteobacteria
nme	*Neisseria meningitidis* MC58	1084	1557	β-proteobacteria
pae	*Pseudomonas aeruginosa* PA01	2004	4168	γ-proteobacteria
**Lactobacillus**				
lbh	*Lactobacillus buchneri*	964	1438	Firmicutes
lbr	*Lactobacillus brevis*	846	1305	Firmicutes
lfe	*Lactobacillus fermentum* IFO 3956	921	1377	Firmicutes
lru	*Lactobacillus reuteri* SD2112	863	1294	Firmicutes
lsn	*Lactobacillus sanfranciscensis*	539	775	Firmicutes
lai	*Lactobacillus acidophilus* 30SC	720	1051	Firmicutes
lay	*Lactobacillus amylovorus* GRL1118	659	897	Firmicutes
lcr	*Lactobacillus crispatus*	719	1071	Firmicutes
lga	*Lactobacillus gasseri*	648	934	Firmicutes
lhl	*Lactobacillus helveticus* H10	667	1005	Firmicutes
ljo	*Lactobacillus johnsonii* NCC 533	670	982	Firmicutes
lke	*Lactobacillus kefiranofaciens*	728	1107	Firmicutes
**Prochlorococcus and Synechococcus**				
pma	*Prochlorococcus marinus* SS120	1061	1480	Cyanobacteria
pmc	*Prochlorococcus marinus* MIT 9515	1030	1463	Cyanobacteria
pmi	*Prochlorococcus marinus* MIT9312	1058	1463	Cyanobacteria
pmm	*Prochlorococcus marinus* MED4	1052	1467	Cyanobacteria
pmn	*Prochlorococcus marinus* NATL2A	1048	1448	Cyanobacteria
syc	*Synechococcus elongatus* PCC6301	1233	1708	Cyanobacteria
syd	*Synechococcus sp.* CC9605	1233	1737	Cyanobacteria

sye	*Synechococcus sp.* CC9902	1167	1630	Cyanobacteria
syf	*Synechococcus elongatus* PCC7942	1278	1790	Cyanobacteria
syr	*Synechococcus sp.* RCC307	1209	1696	Cyanobacteria
syw	*Synechococcus sp.* WH8102	1180	1674	Cyanobacteria
syx	*Synechococcus sp.* WH7803	1269	1762	Cyanobacteria
**Green sulfur bacteria and Green nonsulfur bacteria**				
atm	*Anaerolinea thermophila*	1057	1639	Green nonsulfur bacteria
cap	*Caldilinea aerophila*	1409	2276	Green nonsulfur bacteria
cau	*Chloroflexus aurantiacus*	1526	2474	Green nonsulfur bacteria
det	*Dehalococcoides ethenogenes*	694	1023	Green nonsulfur bacteria
dly	*Dehalogenimonas lykanthroporepellens*	744	1048	Green nonsulfur bacteria
hau	*Herpetosiphon aurantiacus*	1414	2347	Green nonsulfur bacteria
rrs	*Roseiflexus sp.* RS-1	1532	2529	Green nonsulfur bacteria
sti	*Sphaerobacter thermophilus*	1377	2340	Green nonsulfur bacteria
tro	*Thermomicrobium roseum*	1251	2084	Green nonsulfur bacteria
cch	*Chlorobium chlorochromatii*	1073	1547	Green sulfur bacteria
cte	*Chlorobaculum tepidum*	1089	1520	Green sulfur bacteria
cts	*Chloroherpeton thalassium*	1143	1714	Green sulfur bacteria
paa	*Prosthecochloris aestuarii*	1139	1655	Green sulfur bacteria
plt	*Pelodictyon luteolum*	1043	1485	Green sulfur bacteria
