# Supplementary figures and images for: Reconstruction of phyletic trees by global alignment of multiple metabolic networks
Source: BMC Bioinformatics. 2013 Jan 21;14(Suppl 2):S12. doi: 10.1186/1471-2105-14-S2-S12 (PMC3549807; doi:10.1186/1471-2105-14-S2-S12)

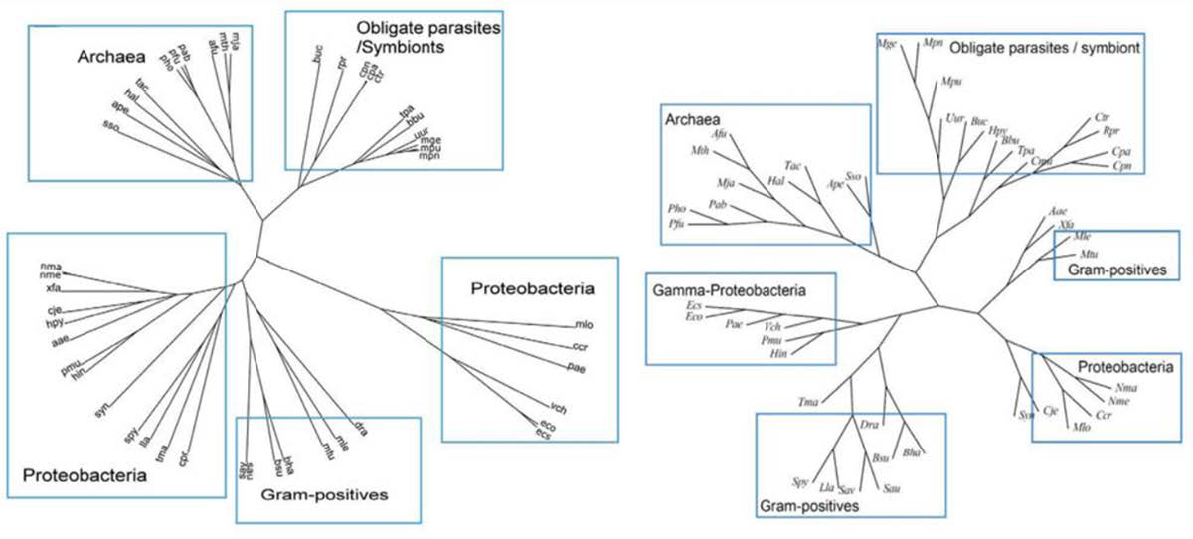

Supplement: Additional file 2 — Comparison of reconstructed phylogenic trees. Left: Reconstruction by Chang et al. [17]. Right: Reconstruction by Zhang et al. [12]. Reprinted under the BioMed Central Open License agreement (BMC Bioinformatics). [file 1471-2105-14-S2-S12-S2.png]

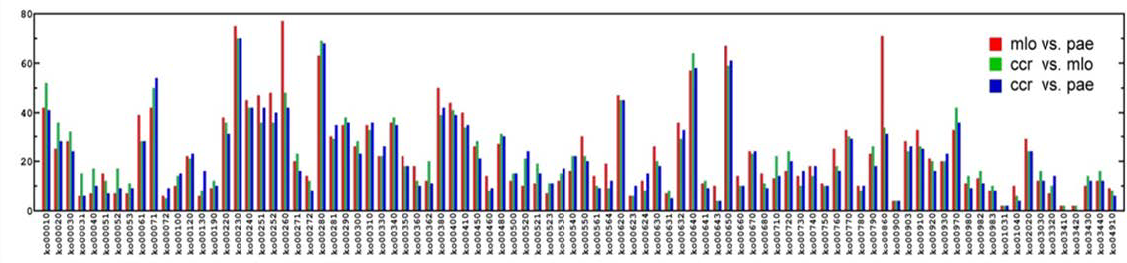

Supplement: Additional file 4 — Statistics for KEGG pathways between three pairs of organisms: (mlo, pae), (ccr, mlo) and (ccr, pae). The x axis represents KEGG pathway IDs, and the y axis represents the number of the constituent enzymes in the pathways. The two pathways ko00260 and ko00860 in the pair (mlo, pae) contain more functional orthologs than those in the pairs (ccr, mlo) and (ccr, pae). [file 1471-2105-14-S2-S12-S4.png]

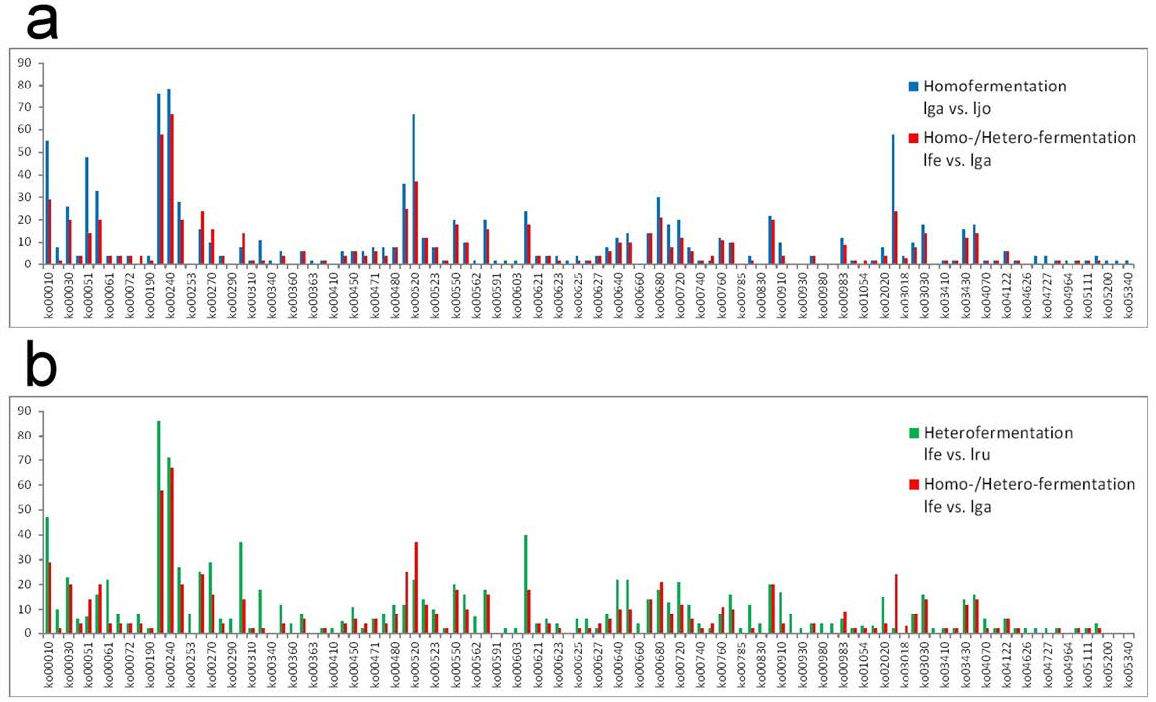

Supplement: Additional file 5 — Statistics for KEGG pathways between two pairs of organisms in Lactobacillus: The x axis represents KEGG pathway IDs, and the y axis represents the number of the constituent enzymes in the pathways. (a) (lga, ljo) in obligate homofermentation, and (lfe, lga) from different fermentation types. (b) (lfe, lru) in obligate heterofermentation, and (lfe, lga) from different fermentation types. [file 1471-2105-14-S2-S12-S5.png]

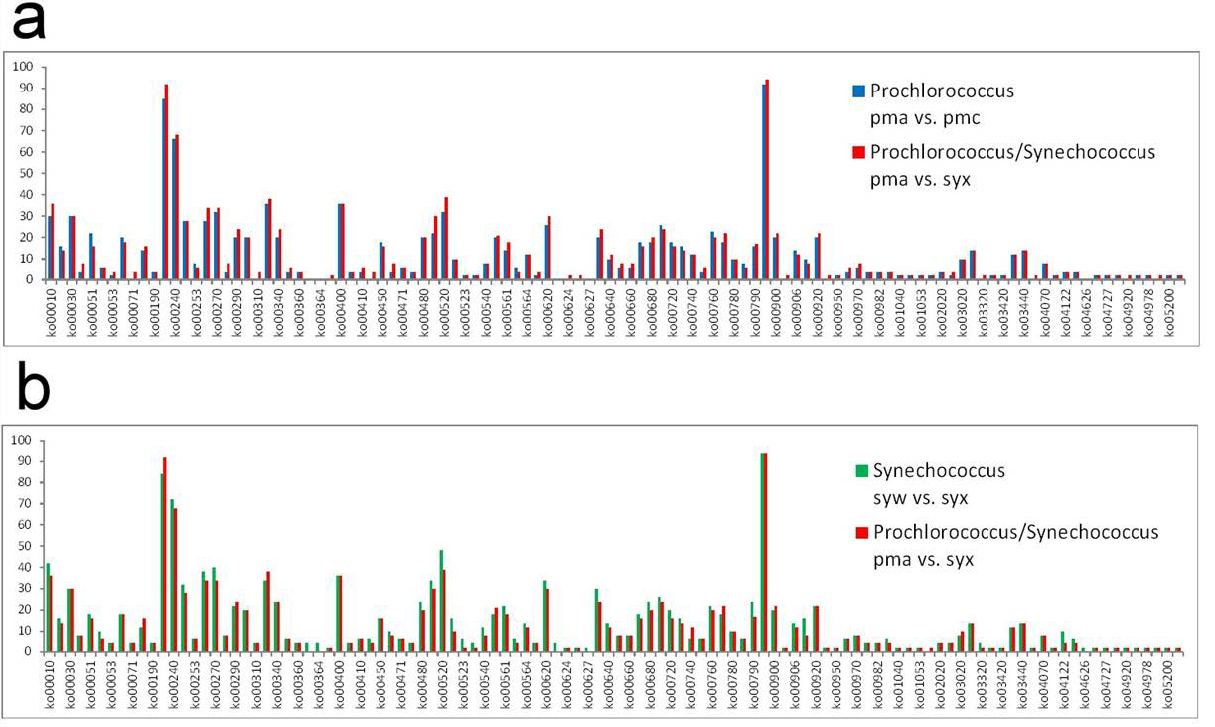

Supplement: Additional file 6 — Statistics for KEGG pathways between two pairs of organisms of Prochlorococcus and Synechococcus: The x axis represents KEGG pathway IDs, and the y axis represents the number of the constituent enzymes in the pathways. (a) (pma, pmc) from Prochlorococcus, and (pma, syx) from Prochlorococcus and Synechococcus, respectively. (b) (syw, syx) from Synechococcus, and (pma, syx) from Prochlorococcus and Synechococcus, respectively. [file 1471-2105-14-S2-S12-S6.png]
