# Supplementary material for: Reconstruction of phyletic trees by global alignment of multiple metabolic networks
Source: BMC Bioinformatics. 2013 Jan 21;14(Suppl 2):S12. doi: 10.1186/1471-2105-14-S2-S12 (PMC3549807; doi:10.1186/1471-2105-14-S2-S12)
Supplement: Additional file 3 — The IsoRankN algorithm. [file 1471-2105-14-S2-S12-S3.pdf]

The IsoRankN Algorithm

The score R_{ij}integrates sequence similarity with network topology similarity for an enzyme pair (i, j), where i and j are from different species. The sequence similarity between each pair of enzymes is their BLAST Bit score [31], whereas the enzyme-enzyme relational overlap represents their topological similarity. More precisely, let α be a parameter that controls the factor of topological similarity relative to sequence similarity$0 \leq \alpha \leq 1$; then, the computation of R can be reduced to an eigenvalue problem and solved by the power method [30].

$$\mathrm{R} = \alpha \mathrm{AR} + (1 - \alpha)E$$

where

$$A_{ij,uv} = \begin{cases} \frac{1}{|N(u)||N(v)|}, if\ u \in N(i)\ and\ v \in N(j), \\ 0, otherwise \end{cases}$$

The topology similarity score for each enzyme pair (i, j) is provided by the possible matches between metabolic neighbors of *i* and *j*. If the enzyme relations (i, u) and (j, v) exist, each of the possible matches comes from $\mathrm{N}(u)$ and $\mathrm{N}(v)$ and shares the score equally, where $\mathrm{N}(u)$ and $\mathrm{N}(v)$ are the neighborhoods of *u* and *v*, respectively.

The star-aligned method yields a more comprehensive understanding between multiple organisms, and the personalized partitioning technique identifies the functionally conserved reaction cluster with respect to each enzyme. The algorithm selects an arbitrary remaining metabolic network and spectrally identifies the conserved reaction cluster for each enzyme. The highly function-related clusters are merged if necessary. The process is repeated until all

enzymes are assigned to a cluster. Note that the software tool is error tolerant and computationally efficient because both of the steps are based on spectral techniques.
